# Risk of Parenteral Nutrition in Neonates—An Overview

**DOI:** 10.3390/nu4101490

**Published:** 2012-10-16

**Authors:** Walter Zingg, Maren Tomaske, Maria Martin

**Affiliations:** 1 Infection Control Programme, University of Geneva Hospitals, rue Gabrielle Perret-Gentil 4, 1211 Genève 14, Switzerland; 2 Neonatology Unit, Department of Pediatrics, Stadtspital Triemli, Birmensdorferstrasse 497, 8055 Zürich, Switzerland; Email: maren.tomaske@triemli.zuerich.ch; 3 Division of Infection Control and Hospital Epidemiology, Department of Environmental Health Sciences, University Medical Center Freiburg, Breisacher Str. 115b, 79106 Freiburg, Germany; Email: maria.martin@uniklinik-freiburg.de

**Keywords:** newborn, preterm, neonate, healthcare-associated infection, parenteral nutrition, bloodstream infection, intravenous catheter, compounded formula, ready-to-use

## Abstract

Healthcare-associated infections (HAI) in preterm infants are a challenge to the care of these fragile patients. HAI-incidence rates range from 6 to 27 infections per 1000 patient-days. Most nosocomial infections are bloodstream infections and of these, the majority is associated with the use of central venous catheters. Many studies identified parenteral nutrition as an independent risk factor for HAI, catheter-associated bloodstream infection, and clinical sepsis. This fact and various published outbreaks due to contaminated parenteral nutrition preparations highlight the importance of appropriate standards in the preparation and handling of intravenous solutions and parenteral nutrition. Ready-to-use parenteral nutrition formulations may provide additional safety in this context. However, there is concern that such formulations may result in overfeeding and necrotizing enterocolitis. Given the risk for catheter-associated infection, handling with parenteral nutrition should be minimized and the duration shortened. Further research is required about this topic.

## 1. Introduction

There has been an increase of preterm births (<37 weeks of gestation) in most countries worldwide over the past years [1]. These patients are at risk for healthcare-associated infections (HAIs), in particular very low birth weight (VLBW, <1500 g birth weight) and extremely low birth weight (ELBW, <1000 g birth weight) infants. They require longer care in neonatology units, are exposed to more invasive devices, and have an immature immune system, as well as low levels of transplacentally acquired antibodies. Reported HAI-rates in neonatal care range from 6 to 27 per 1000 patient-days [2,3,4,5,6,7] and thus are higher than in other patient groups. Large cohort studies reported high mortality and impaired neurological development in neonates after HAI [8,9]. A study among 1051 neonates in a Brazilian neonatal intensive care unit (NICU) identiﬁed risk factors for HAI to include mechanical ventilation, inappropriate fraction of inspired oxygen, and duration of central venous catheterization [10].

Most nosocomial infections are due to bloodstream infections (BSIs) representing up to more than three-quarters of all HAI among neonates [7,11,12,13,14,15,16,17]. Most BSIs are associated with catheter use, in particular central lines such as peripherally inserted central catheters (PICC) and umbilical lines [18]. A total of 205 neonates out of 2935 neonates in two NICUs in New York developed BSIs after a mean of 21.4 and 35.7 days of life in each NICU, respectively [19]. Risk factors included low birth weight (with an additional 9% risk for each 100 g weight decrease), the presence of a central venous catheter (9.3-fold risk increase when compared to children without a catheter) and total parenteral nutrition (RR: 4.7). A Swiss study identified ELBW [OR: 2.24 (1.42–3.53); *p* < 0.001] and the use of parenteral nutrition (PN) [OR: 2.23 (1.10–4.55); *p* = 0.027] as the only two independent risk factors for BSI over 8 years [18]. Median time-to-infection was seven days. 

Central line-associated bloodstream infections (CLABSIs) extend hospital length of stay by an average of 7 days and result in attributable costs of 3700 to 29,000 USD per infection [20]. A case-control study estimated extra-costs per neonate to 10,440 USD with 5.2 extra hospital days [21]. BSI-attributed costs are particularly high in Candida infections, especially in ELBW infants [22]. 

## 2. Growth of Microorganisms in Parenteral Nutrition

### 2.1. Microorganisms in Parenteral Nutrition

Various *in vitro* studies tested the growth of micro-organisms in single compounds of PN, in total parenteral nutrition (TPN), or in total nutrient admixtures (TNA). Testing of 12 different pathogens (*Staphylococcus epidermidis*, *Staphylococcus aureus*, *Enterobacter cloacae*, *Klebsiella oxytoca*, *Serratia marcescens*, *Acinetobacter calcoaceticus*, *Stenotrophomonas maltophilia*, *Pseudomonas aeruginosa*, *Burkholderia cepacia*, *Flavobacterium* spp., *Staphylococcus saprophyticus*, and *Candida albicans*) in a representative TNA of 17.6% glucose, 5% amino acids, and 4% lipid (pH 5.6, osmolality 1778) compared to a control solution of 5% dextrose in water at 4 °C, 25 °C, and 35 °C only grew *C. albicans* and *S. saprophyticus* in TNA at 25 °C and 35 °C and only after 24 to 48 h. The authors concluded that TNA was a poor growth medium for most nosocomial pathogens [23]. While lipid emulsion and broth grew all tested organisms (*Escherichia coli*, *Enterobacter cloacae*, *P. aeruginosa*, *S. aureus*, and *C. albicans*) in another study, only *C. albicans* was found to proliferate in TPN [24]. *Candida albicans* demonstrated significant growth regardless of fat contents (0% or 5%) in admixtures containing variable concentrations of dextrose in an *in vitro* study [25]. Gram-negative microorganisms such as *Klebsiella pneumoniae*, *E. coli*, and *P. aeruginosa* were able to proliferate in TNA with glucose, amino acids, and lipid emulsion, but growth was impaired in conventional TPN without lipids [26]. *S. epidermidis* was not able to proliferate in any admixture tested; however, *C. albicans* grew well in all admixtures. A recent study showed that the proliferation of *S. epidermidis* was not only affected by adding lipids to TPN, but also depended on glucose concentration and total non-nitrogen energy [27]. Growth was also reduced by higher pH values (~8.4). An older study, on the other hand, found considerable growth of a number of Gram-positive and Gram-negative microorganisms in TPN without lipids [28]. The same study looked at growth in catheters challenged with bacteria and flushed with TPN for three days, but did not find significant increase of growth over time.

### 2.2. Growth of Microorganisms on Catheters Used for Parenteral Nutrition

*Staphylococcus epidermidis* was cultured from 7 out of 9 catheters after lipid infusion but only from 3 out of 13 catheters after glucose-infusion (*p* = 0.016) in a rabbit model [29]. Lipid but not glucose solutions containing low protein levels (0.1%–1.0%) supported the survival and growth of *S. epidermidis*. The reason for enhanced growth in the context of lipid administration is not clear. A modulation of the proinflammatory cytokine response to *Staphylococcus epidermidis* by lipids has been suggested [30]. In a *S. epidermidis* sepsis model of whole cord blood cells from healthy infants, IL-6, IL-8, and TNF-α expression of CD14+ cells was signiﬁcantly enhanced upon addition of a 1% lipid formulation, while lower lipid concentrations had no remarkable effect. When glucose was added to whole cord blood cultures, a dose-dependent effect was demonstrated for IL-8 expression but not for other cytokines.

## 3. Sepsis in Neonates

Group B *Streptococci* were predominant (47%), followed by *E. coli* (23%), *Staphylococcus* spp. (13%) and Gram-negative rods other than *E. coli* (8%) in early-onset sepsis (sepsis in the first three days of life) in a long term surveillance of a US NICU [31]. The most common organisms in late-onset sepsis (>3 days after birth) were coagulase-negative *Streptococci* (CoNS) (39%), followed by *Escherichia coli* (9%) and *C. albicans* (9%) [31]. This finding was confirmed by others, with nearly all isolated CoNS (87%) being methicillin resistant [32].

## 4. Parenteral Nutrition Is a Risk for Late-Onset Sepsis

There is evidence that parenteral nutrition is associated with BSI. Nosocomial BSI due to parenteral nutrition is a potentially fatal complication with an attributable mortality rate of 11% in neonates [33]. High mortality, high incidence densities of late-onset-sepsis and CLABSI, require efforts to reduce this risk as much as possible [2,18,33,34,35]. The reasons for parenteral nutrition being identified as a risk factor by a number of studies include contamination of infusates; however, a catheter may infect at any time during catheterization due to handling, which is exemplified by the fact that CoNS is the most common pathogen identified in neonatal CLABSI [2].

### 4.1. Australia

TPN-use was found to be a significant risk factor among preterm neonates in a small single center study in Australia [OR (CI 95%): 4.17 (1.37–12.7)] [36]. 

### 4.2. Brazil

A retrospective study in Brazil among 948 neonates found parenteral nutrition significantly associated with HAI [OR (CI 95%): 6.35 (4.14–9.75); *p* < 0.01] [37].

### 4.3. Canada

A large Canadian study reported the results of a neonatal network of 16,538 infants [38]. HAI was detected in 23.5% (765/3253) of VLBW infants and in 2.5% (329/13,244) of infants >1500 g. Multiple logistic regression analysis disclosed parenteral nutrition being a significant risk factor for neonates <1500 g [OR (CI 95%): 3.9 (3.0–5.2)] and >1500 g [OR (CI 95%): 5.1 (3.8–6.9)].

### 4.4. Colombia

A Colombian group investigated risk factors for BSI due to *Candida* spp. in a single center. Prolonged hospitalization (*p* < 0.05), missing prenatal birth control (*p* < 0.05), and parenteral nutrition (*p* < 0.05) were associated with BSI due to *Candida* spp. [39].

### 4.5. Denmark

Six hundred and eighty three neonates were included in a prospective single center study in Denmark. The overall incidence densities of HAI and BSIs were 8.8/1000 patient-days and 5.1/1000, respectively [40]. Parenteral nutrition was found an independent and significant risk factor for BSIs [HR (CI 95%): 2.71 (1.27–5.79); *p* = 0.01]. 

### 4.6. Italy

A multicenter cohort study in six Italian NICUs reported an overall HAI incidence density of 6.9/1000 patient-days [41]. Administration of parenteral nutrition [HR (CI 95%): 8.1 (3.2–20.5)] also was found an independent risk factor for HAI in neonates >1500 g. 

### 4.7. Mexico

Similar results were reported by Avila-Figueroa and colleagues (Mexico) who looked into BSI due to CoNS [42]. They found that lipids were independently associated with subsequent BSI due to CoNS among other procedures at risk [OR (CI 95%): 9.4 (1.2–74.2)]. 

### 4.8. Spain

The overall rate of HAI in the NICU was 27/1000 patient-days in a Catalan study [7]. The most significant predisposing risk factors for HAI were birth weight <1000 g [RR (CI 95%): 2.8 (1.0–8.0)], umbilical arterial catheterization [RR (CI 95%): 5.7 (1.1–28.5)] and parenteral nutrition [RR (CI 95%): 2.4 (1.2–4.6)]. 

### 4.9. Saudi Arabia

A single center study in Saudi Arabia reported total parenteral nutrition [OR (CI 95%): 5.62 (2.78–11.35)] significantly associated with HAI in a cohort of 401 neonates [43]. The overall incidence density of nosocomial infections in this study was 13.7/1000 patient-days. 

### 4.10. Switzerland

Parenteral nutrition was an independent and significant risk factor for bacteraemia and clinical sepsis [HR (CI 95%): 2.23 (1.10–4.55); *p* = 0.027] in a prospective single center study in Switzerland [18]. The follow-up was eight years and included 1124 neonates exposed to a central line, such as a peripherally inserted central catheter (PICC) or an umbilical line.

### 4.11. The Netherlands

A prospective surveillance study over three years in a university medical center in the Netherlands enrolled 762 neonates [44]. The BSI-incidence density was 14.9/1000 patient-days. The main risk factors for BSI were birth weight [HR (CI 95%): 1.79 (1.45–2.17)] and parenteral feeding with an all-in-one mixture produced by the in-hospital pharmacy [HR (CI 95%): 3.69 (2.03–6.69)].

### 4.12. United Kingdom

A single center study in a Level-II NICU in the United Kingdom revealed an overall sepsis rate of 77 per 1000 NICU admissions in 1612 neonates [45]. Administration of TPN was the leading risk factor for late-onset sepsis (88%).

A prospective cohort study in a NICU in England over a study period of almost two years included 1367 neonates with a mean (±SD) gestational age of 31 weeks (±4.4) and birth weight of 1607 g (±817 g) [46]. Significant adjusted risk factors were gestational age <26 weeks [IRR (CI 95%): 2.5 (1.7–3.8)] and the use of parenteral nutrition, whether administered centrally or peripherally [IRR (CI 95%): 14.2 (8.8–22.9); *p* < 0.001].

### 4.13. United States

A retrospective cohort study in the US did not identify specific genetic associations among twins, but the duration of TPN was found an independent risk factor for late-onset sepsis [Coeff (CI 95%): 0.041 (0.017–0.064); *p* < 0.001] [47]. The same author tested risk factors for *S. marcescens* BSI in neonates in a matched case-control study. Interestingly, an association with parenteral nutrition was found for this pathogen only when the cohort was compared to neonates with *E. coli*-BSI [OR (CI 95%): 3.27 (1.20–8.92); *p* = 0.02] but not when compared to neonates without BSI [48].

Infants with CoNS-BSI were 5.8 times (CI 95%: 4.1–8.3) as likely as those in the control group to have received intravenous lipid emulsion before the onset of bacteraemia in a case-controlled study including 882 infants treated in two NICUs in the USA [49].

The differences in the risk of PICC-placement in the upper as compared to the lower extremities was investigated in a large preterm population in the USA [median gestational age and weight: 28 (CI 95%: 25.5–30); 937 (CI 95%: 760–360)] [50]. The incidence densities of the upper and lower extremities were 7.1/1000 catheter-days and 4.8/1000, respectively. However, administration of lipid-containing parenteral nutrition was significantly longer (46 days) in neonates with catheter-related BSI (CRBSI) as compared to neonates without CRBSI (25 days; *p* < 0.01).

## 5. Outbreaks Due to Contaminated Parenteral Nutrition

Various outbreaks due to contaminated parenteral nutrition are reported in the literature. Outbreaks vary in size, time span, and pathogens. Interestingly, most outbreaks are due to contamination by Gram-negative bacteria (Table 1). The most likely time point for contamination appears to be within the span of PN-preparation. For yeasts however, days of TPN-administration seems to be more predictive [51,52]. Two outbreaks among adults due to contaminated TPN-products further illustrate the potential harm of contaminated preparations: A multistate outbreak of *S. marcescens* BSIs linked to contaminated MgSO4 that was distributed nationally by a compounding pharmacy [53]. The outbreak included 18 confirmed and 7 probable adult cases in California and New Jersey. Another outbreak due to *S. marcescens* involved 19 patients in six hospitals in Alabama, US. Nine deaths were related to contaminated parenteral nutrition preparations from a compounding pharmacy. The identical strain of *S. marcescens* was cultured from a tap water faucet in the pharmacy which was used to rinse production equipment [54]. Other outbreaks have been described (Table 1), most of them confirmed by pulsed-field gel-electrophoresis [55,56,57,58,59], ribotyping [60], or by an identical antibiotic susceptibility testing [61].

**Table 1:** Published outbreaks among neonates related to parenteral nutrition.

Author	Ref.	Pathogens	*n*	Deaths	Confirmation ^1^	PN-preparation	Most likely way of contamination
Maltezou	[62]	*Serratia marcescens*	57	9	Epidemiology	On ward	Preparation
Arslan	[55]	*Serratia marcescens*	7	0	Culture/PFGE	On ward	Preparation
Bou	[56]	*Leuconostoc mesenteroides *	11 (42)	3 of 42	Epidemiology	Hospital pharmacy	Preparation
					Culture/PFGE		
Campos	[57]	*Enterobacter hormaechei*	19	ND	Culture/PFGE	Manufacturer	Preparation
De Vegas	[58]	*Acinetobacter *RUH 1139	24	ND	Culture/PFGE	Hospital pharmacy	Handling on ward
Perniola	[52]	*Rhodotorula mucilaginosa*	4	0	Epidemiology	Not specified	Not specified
Habsah	[61]	*Pantoea *spp.	8	7	Culture/AB	Hospital pharmacy	Preparation
Doit	[60]	*Burkholderia cepacia*	8	ND	Culture/Ribotype	manufacturer	Contaminated rubber stoppers
Aragao	[51]	*Pichia anomala*	4	0	Epidemiology	Hospital pharmacy	Handling on ward
Tresoldi	[59]	*Enterobacter cloacae*	11	7	Culture/PFGE	Hospital pharmacy	Preparation
Archibald	[63]	*Enterobacter cloacae *&* Pseudomonas aeruginosa*	6	2	Epidemiology	On ward	Preparation

ND: not determined; ^1^ Confirmation of the pathogen is done either by PFGE (pulsed-field gel-electrophoresis [PFGE]; ribotyping, identical antibiotic susceptibility testing [AB]), or by an epidemiological association.

## 6. Compounding Is a Risk for Contamination

If complicated transfers of the medium from vials and ampoules to intravenous bags are performed, even with stringent aseptic technique, the TNA-contamination rate is estimated at about 5.2% [64]. Although performed under laminar flow by trained personnel in a pharmacy, transfer sets for compound pumps showed growth after 24 h with skin contaminants [65]. A clinical-oriented study looking at differential methods of neonatal intravenous fat emulsion preparation found contamination in 3.3% when emulsions were repackaged in syringes before use [66]. In a Mexican NICU-study mixtures made by nurses were more likely to be contaminated than commercial preparations [OR (CI 95%): 3.1 (1.1–8.5); *p* = 0.037] [67].

Individual tailored TNA should be prepared every day with strict aseptic technique in the pharmacy, not in the ward, and stored in a refrigerator at 4 °C [68]. The solutions prepared in such a way are stable for 96 h. Current practice standards require parenteral preparations to be compounded in a clean room to minimize microbial contamination. Proper storage, refrigeration, and infusion time of no more than 24 h reduce the chance of microbial contamination or growth in parenteral nutrition formulation [68]. Minimum requirements for compounding vary from country to country but mostly include the use of a Class A/Class 100 laminar flow cabinet either operated in a Class B/Class 1000 or a relatively uncontrolled environment [69].

## 7. Ready-to-Use Formulation in Preterm Infants

Large hospitals perform pharmacy compounding to all-in-one admixtures rather than using commercial formula in compartment bags. While in adults, ready-to-use admixtures have become standard, most parenteral nutrition preparations for neonates are still compounded in local pharmacies [70,71]. In adults, ready-to-use multichamber bags containing three sterilized macronutrient solutions in separate chambers of a single, closed plastic system are widely available and have been used for many years [72,73]. Standardized solutions can be also used in infants once they tolerate mild or moderate variations in nutritional intake [74,75,76,77,78,79]. However, there are still concernsabout ready-to-use preparations in preterms. Many neonatologists consider customized admixtures prepared by the pharmacy superior to ready-to-use preparations to provide optimal nutrition although standardized parenteral nutrition solutions in combination with early promotion of enteral feeding with human milk have been associated with improved nutritional support in very preterm infants [79,80].

## 8. Conclusions

Healthcare associated infections are a permanent challenge in neonates. In particular, preterm infants are at risk for infection due to an immature immune system and lower levels of transplacental antibodies. The majority of nosocomial infections are due to CLABSI, which are associated with prolonged hospital stay causing significant attributable costs. TPN and TNA have been identified as independent risk factors for HAI, BSI or sepsis by many studies. The body of evidence is uncontested and robust. These findings have a strong pathophysiological background as TPN with lipids promote the growth of a wide spectrum of microorganisms that is even further increased by human serum. In particular, *Candida* species can grow rapidly in almost all TPN solutions regardless of acidity and lipid content. Some bacterial species may grow in lipid-containing TNA unless the pH-value is extreme [81], but they poorly grow in parenteral nutrition without lipids due to the acidity of the solution [82].

Maintaining sterility of nutritional admixtures for parenteral use upon preparation is of utmost importance. Regulations and recommendations have been issued to assure the quality of the final product. Despite compounding in the pharmacy under strict aseptic technique, outbreaks due to contaminated admixtures have been reported. Given the high CLABSI incidence in neonates associated with high attributable mortality, further prevention efforts are required. Although many neonatologists still have concerns about the suitability of ready-to-use preparations on nutritional levels, such products may offer a better safety profile in terms of infections. The risk for CLABSI through prolonged catheterization and parenteral nutrition might be further reduced by encouraging enteral feeding. Current data do not provide evidence that delayed induction of progressive enteral feeding or slow advancement of enteral feed volumes reduces the risk of NEC in VLBW infants. On the other hand, delaying induction and increasing the volume of enteral feeds at slower rather than faster rates results in several days delay in regaining birth weight and establishing full enteral feeds [83,84]. Rapid advancement of enteral feeding may reduce the duration of parenteral nutrition and catheter dwell time and, thus, such an approach may be favored. It was not the scope of the review to comment on the risks and benefits of accelerated enteral feeding. However, a recent randomized controlled trial suggests that such a strategy may even contribute to the prevention of HAI and in particular of CLABSI and clinical sepsis [85].
